# The GRAIDS Trial: a cluster randomised controlled trial of computer decision support for the management of familial cancer risk in primary care

**DOI:** 10.1038/sj.bjc.6603897

**Published:** 2007-08-14

**Authors:** J Emery, H Morris, R Goodchild, T Fanshawe, A T Prevost, M Bobrow, A L Kinmonth

**Affiliations:** 1 General Practice, School of Primary, Aboriginal and Rural Health Care, University of Western Australia, 328 Stirling Highway, Claremont, Western Australia 6010, Australia; 2 General Practice & Primary Care Research Unit, University of Cambridge, Institute of Public Health, Robinson Way, Cambridge CB2 2SR, UK; 3 General Practice & Primary Care Research Unit, University of Cambridge; MRC Biostatistics Unit, University of Cambridge, Institute of Public Health, Robinson Way, Cambridge CB2 2SR, UK; 4 Department of Medical Genetics, University of Cambridge, Addenbrookes NHS Hospital Trust, Cambridge, UK

**Keywords:** primary health care, clinical decision support systems, neoplastic syndromes, hereditary, randomised controlled trials

## Abstract

The objective was to evaluate the effect of an assessment strategy using the computer decision support system (the GRAIDS software), on the management of familial cancer risk in British general practice in comparison with best current practice. The design included cluster randomised controlled trial, and involved forty-five general practice teams in East Anglia, UK. Randomised to GRAIDS (Genetic Risk Assessment on the Internet with Decision Support) support (intervention *n*=23) or comparison (*n*=22). Training in the new assessment strategy and access to the GRAIDS software (GRAIDS arm) was conducted, compared with an educational session and guidelines about managing familial breast and colorectal cancer risk (comparison) were mailed. Outcomes were measured at practice, practitioner and patient levels. The primary outcome measure, at practice level, was the proportion of referrals made to the Regional Genetics Clinic for familial breast or colorectal cancer that were consistent with referral guidelines. Other measures included practitioner confidence in managing familial cancer (GRAIDS arm only) and, in patients: cancer worry, risk perception and knowledge about familial cancer. There were more referrals to the Regional Genetics Clinic from GRAIDS than comparison practices (mean 6.2 and 3.2 referrals per 10 000 registered patients per year; mean difference 3.0 referrals; 95% confidence interval (CI) 1.2–4.8; *P*=0.001); referrals from GRAIDS practices were more likely to be consistent with referral guidelines (odds ratio (OR)=5.2; 95% CI 1.7–15.8, *P*=0.006). Patients referred from GRAIDS practices had lower cancer worry scores at the point of referral (mean difference −1.44 95% CI −2.64 to −0.23, *P*=0.02). There were no differences in patient knowledge about familial cancer. The intervention increased GPs' confidence in managing familial cancer. Compared with education and mailed guidelines, assessment including computer decision support increased the number and quality of referrals to the Regional Genetics Clinic for familial cancer risk, improved practitioner confidence and had no adverse psychological effects in patients. Trials are registered under N0181144343 in the UK National Research Register.

Clinical translation of advances in understanding the genetics of common disease will require primary care practitioners to play an increasing role in providing genetic advice (Qureshi *et al*, 2004). Cancer genetics provides a model for the genetics of common disease and their clinical implications for primary care (Emery *et al*, 2001). The discovery of genes that place individuals at increased risk of breast, ovarian and colorectal cancer has had important and immediate clinical applications (Wooster and Weber, 2003). Key tasks for primary care practitioners include identifying individuals likely to be at increased genetic risk and advising those for whom genetic testing and increased disease surveillance offer little benefit. Previous audits of referrals to familial cancer clinics in the UK show that approximately 30% of referrals from general practice are for patients whose risk is not significantly raised on current evidence (Wonderling *et al*, 2001).

Previously we reported experimental and qualitative evaluations of a prototype computer decision support tool for the management of familial cancer risk in primary care (Emery *et al*, 1999, 2000). These demonstrated the functionality and design of the software, and also demonstrated its potential to improve general practitioners' management decisions in simulated cases. This work underpinned the development of the GRAIDS software (Genetic Risk Assessment on the Internet with Decision Support) (Emery, 2005). We now report the results of a randomised controlled trial of an assessment strategy using the GRAIDS software in British general practice compared with best current practice.

## MATERIALS AND METHODS

### Design

This was a pragmatic cluster randomised controlled trial with randomisation, at the level of the general practice, to GRAIDS or current ‘best practice’ (Campbell *et al*, 2000). Outcomes were measured at practice, practitioner and patient levels. Within the intervention group, we used an exploratory design with fixed and adaptive arms based on a threshold of software use.

### Objectives

We hypothesised that the new assessment strategy (GRAIDS) would result in a greater proportion of referrals to the Regional Genetics Clinic that were consistent with the risk assessment guidelines for familial breast/ovarian cancer and familial colorectal cancer, than current best practice. Secondary hypotheses included that patients from intervention practices would have greater knowledge about familial cancer without higher cancer worry, at the point of referral than from comparison practices. Within the GRAIDS practices, we predicted that patients who were not referred would have lower risk perception and lower cancer worry than those who had been referred, and that the intervention would increase practitioner confidence in management.

#### Participant practice teams

We invited 170 general practice teams in the Eastern Region of England, with a minimum of three full-time-equivalent doctors, to join the trial. Inclusion criteria were that the practice was connected to the health service intranet (NHSnet) and referred patients with a family history of cancer to the Eastern Regional Genetics Clinic at Addenbrookes Hospital NHS Trust, Cambridge. Forty-five practice teams agreed to participate and were randomised to GRAIDS (intervention) or best practice (comparison) strategies.

#### Interventions and recruitment of patients

*Intervention; GRAIDS strategy*: All general practitioners and practice nurses attended a 45-min educational session on cancer genetics, delivered at their general practice. They were also introduced to the principles of the GRAIDS intervention. Each practice team selected a single clinician (general practitioner or practice nurse) to act as the ‘lead clinician’ to manage all patients who expressed concerns about their family history of breast or colorectal cancer. In larger practices, this role could be shared by two clinicians. The lead clinicians attended a further 90-min interactive training session to learn to use the GRAIDS software.

The GRAIDS software links a user-friendly pedigree-drawing tool to patient-specific management advice regarding a family history of breast/ovarian and colorectal cancer, and provides additional numerical risk information about breast cancer (Supplementary Figure 1). The software applies Cyrillic technology (Benson, 2000) to create pedigrees and assesses familial cancer risk using two parallel methods: the implementation of risk assessment guidelines and an epidemiological risk model. In the GRAIDS Trial, we implemented the regional guidelines for familial breast/ovarian cancer and familial colorectal cancer (Table 1). In addition, the Claus model (Claus *et al*, 1991) was applied to provide breast cancer risk information in a range of verbal and graphical modes. The regional guidelines are principally designed to assess cancer risk and categorise people into increased risk or population risk; the familial breast/ovarian cancer guidelines additionally categorise women into moderate and high risk, the latter representing people who are also at clinically significant risk of carrying a BRCA1/2 mutation. The guidelines are used to inform referrals for those at increased risk of cancer to the Regional Genetics Clinic.

The GRAIDS software operates on a central server within a computer network. In this trial, the software was installed on a secure server at the Addenbrookes Hospital NHS Trust. Each lead clinician accessed the GRAIDS software via their NHSnet connection, using a practice-specific password.

Patients were invited to participate if they expressed concerns about their family history of breast or colorectal cancer in a consultation. They were referred to the lead clinician and given a detailed explanation of project participation, and a family history questionnaire to complete before the next consultation. The family history questionnaire was designed as part of the GRAIDS strategy to improve the accuracy of the family history information provided by participants.

*Fixed and adaptive sub-groups*: Within the intervention arm, practices were randomised to either a fixed or adaptive sub-group. In the adaptive group, the lead clinician was interviewed 3 months after training, if frequency of software usage was below a predefined level based on size of the practice population. The interview identified reasons for low usage and aimed to resolve any problems using the software. In the fixed group, practices received the intervention as described above, with no opportunity for additional input. The purpose of this was to include the option of additional clinician training or adjustment to the software in the adaptive arm during the trial, to increase software use.

*Comparison; current ‘best practice’*: All general practitioners and practice nurses attended a 45-min educational session on cancer genetics delivered at their general practice (Watson *et al*, 2001). Afterwards, they were each mailed a paper copy of the regional guidelines for familial breast/ovarian cancer and familial colorectal cancer. Patients were unaware that they would be invited to participate in the trial, until they were referred to the Regional Genetics Clinic.

### Measures

#### Randomised comparisons

*Practice level, principal outcome and appropriate referral rates*: We audited all referrals to the Regional Genetics Clinic from trial practices during the study period. ‘Appropriateness’ of referral was defined in two ways: (1) (principal outcome) consistency of the family history reported in the general practitioner's referral letter with the regional guidelines for familial breast/ovarian and colorectal cancer; (2) (secondary outcome) the final expert risk assessment conducted by the Regional Genetics Clinic staff among attending patients, according to current best evidence, to determine if the patient was at significantly increased risk of familial cancer. A ‘relevant referral’ was for the individual person referred, rather than their relative, and about their family history of either breast, ovarian or colorectal cancer.

*Patient level: (referred patients only):* Questionnaires were sent to patients when a referral was received at the Regional Genetics Clinic. The questionnaire measured risk perception, knowledge about familial cancer and cancer worry using disease-specific measures. Items were taken from established instruments identified in a systematic review of genetic counselling for familial cancer (Braithwaite *et al*, 2004). Risk perception was measured on a scale of 1–7, relative to the general population, considering 1 as ‘much less likely…’ and 7 as ‘much more likely to develop breast/bowel cancer than other people of your age’. For one analysis, responses of 1–4 were classified as ‘population risk’ and 5–7 as ‘increased risk’. This was compared to the risk assessment conducted by the Regional Genetics Clinic to classify patients as under-estimators, accurate-estimators and over-estimators (Watson *et al*, 1999).

#### Comparisons within GRAIDS arm only

*Practitioner level*: We measured the frequency of use of the software remotely from server activity. A questionnaire examined the attitudes of lead clinicians towards using the GRAIDS software, their confidence in risk assessment and managing patients with a family history of cancer, problems using the software and intention to continue using it (Braithwaite *et al*, 2002). This questionnaire was provided before the lead clinician training and 2 weeks and 12 months after. Lower scores on these instruments reflected more positive attitudes or greater agreement with a statement. Data on the length of consultation with the lead clinician were obtained from electronic appointment systems in 20 intervention practices.

*Patient level*: Those in the GRAIDS arm who were not referred, were sent questionnaires 2 weeks after their consultation with the lead clinician for comparison with those referred from GRAIDS practices.

### Sample size

Twenty intervention and 20 comparison practices allowed an estimation of effect size on appropriateness of referrals for familial cancer with a precision of ±11% as measured by the 95% confidence interval (CI) width, equivalent to 80% power to detect a 15% difference between arms. Ten practices per intervention arm also provided 80% power to detect a 25% relative difference (adaptive *vs* fixed arms) in use of software at 1 year, and 33% relative difference at 3 months using practice-level *t*-tests at the 5% level of significance. Sample sizes were determined through simulation using S-plus 2000 software (MathSoft Inc., Seattle, WA, USA).

### Randomisation

Practices were randomised using a partial minimisation procedure that dynamically adjusted the randomisation probabilities in order to provide a balance between arms in the mean number of patients aged 20–50 years per practice and to achieve the planned allocation ratio of 1 : 1 : 2 for adaptive intervention, fixed intervention and comparison arms. Randomisation was conducted independently by a statistician (ATP) who had no contact with practices.

### Statistical methods

Rates of software use were calculated, for each practice, as the annual number of software uses per 10 000 registered patients, and were compared between fixed and adaptive arms using a *t*-test. The same method was used to compare referral rates between GRAIDS and comparison arms. The binary outcomes of referral appropriateness were analysed using a generalised linear mixed-effects model, allowing for practice as a random effect in order to account for the cluster randomised design.

Patient questionnaire outcomes were analysed using linear or generalised linear mixed-effects models as appropriate, allowing for practice as a random effect. Numerical knowledge and worry scales were analysed as continuous variables.

Within the GRAIDS arm, practitioner outcomes were analysed using a paired *t*-test to compare attitudes towards using the GRAIDS software 2 weeks and 12 months after training with pre-training levels. A significance level of 5% was used for all tests. All analyses were conducted on an intention-to-treat basis. Analysis was carried out using SPSS version 12.5 and R version 2.0.1.

Ethical and research governance approval for the study were received by the Eastern Multi-Centre Research Ethics Committee and the relevant primary care trusts, respectively. For the intervention arm, participants signed a consent form at the beginning of their consultation with the lead clinician. For the comparison arm, consent was assumed from return of the questionnaire.

## RESULTS

### Practice characteristics

**Figure 1:**
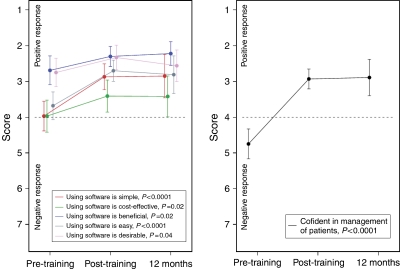
Changes in lead clinicians' attitudes towards the GRAIDS software, and confidence in managing familial cancer over time (mean score with 95% CI shown; *P*-values refer to comparison of pre- and post-training responses).

All 45 practice teams were in the trial for a minimum of 12 months and none withdrew. Twenty-three practice teams were randomised to the intervention and 22 to the comparison group. Table 2 presents the main characteristics of practices and participants in the two trial arms. There were no statistically significant differences in practice characteristics at baseline. The flow of participants through the trial is shown in Figure 1.

One hundred and sixty-two relevant referrals were registered at the Regional Genetics Clinic from GRAIDS practices (162/168 referred) and 84 from comparison practices during the trial. These denominators were used for analyses of referral rates. One hundred and sixty-nine participants had attended their Regional Genetics Clinic appointment by the end of the trial and contributed to the comparison of appropriateness of referral against final Regional Genetics Clinic risk assessment (GRAIDS arm: 117/162 participants, Comparison arm: 52/84 participants). Twenty-two participants were referred for a family history of both breast and colorectal cancer, and therefore contributed two risk assessments in the analyses of appropriateness of referral letters (GRAIDS arm 21; comparison arm 1). Sixteen of these attended the Regional Genetics Clinic within the trial period (GRAIDS arm 15; comparison arm 1). In total, 132 risk assessments in the GRAIDS arm and 53 in the comparison arm were obtained. Questionnaires were returned by 75% of referred participants from the GRAIDS arm and 64% from the comparison arm.

### Randomised comparisons

#### Practice level

Principal outcome: appropriate referral rates: There were 162 relevant referrals made by the GRAIDS practices and 84 referrals by the comparison practices to the Regional Genetics Clinic for familial cancer risk assessment. GRAIDS practice teams referred a mean 6.2 (standard deviation (s.d.), 3.1) per 10 000 registered patients per practice per year compared to 3.2 (s.d., 2.8) per 10 000 registered patients per practice per year in comparison practices (mean difference 3.0; 95% CI 1.2–4.8, *P*=0.002).

A significantly higher proportion of referral letters was consistent with the regional guidelines in the intervention arm than in the comparison arm (breast cancer alone and breast and colorectal cancer combined; Table 3). There was no overall difference between groups in the final risk assessment conducted by staff at the Regional Genetics Clinic. This was due to the large proportion of referrals from GRAIDS practices for family history of colorectal cancer that, while consistent with regional guidelines, on final risk assessment by the Regional Genetics Clinic staff, were deemed to be at population risk. Participants referred from intervention practices about colorectal cancer risk were significantly more likely to be at population risk than those from comparison practices when their risk was determined at the Regional Genetics Clinic, even though the referral letter was consistent with increased risk defined by the familial colorectal cancer guideline. The referrals about colorectal cancer did not account for the significantly higher number of referrals overall from intervention practices.

#### Patient level

There were no significant differences in knowledge scores between patients referred from intervention or comparison practices. Cancer worry scores were significantly lower in patients from intervention practices than those from comparison practices. There was no difference in mean risk perception between patients referred from intervention or comparison practices (Table 4). There was a non-significant trend towards more accurate risk perception at the point of referral in intervention patients with fewer overestimating risk (odds ratio (OR) 1.50, 95% CI 0.62–3.67; *P*=0.36) (Table 5).

### Comparisons within GRAIDS arm only

#### Practitioner level

Software use: The software was used with patients 219 times during the trial; this equates to a mean use of 8.27 per 10 000 registered patients per practice per year. There was no clear trend in frequency of use of the software during the first 12 months of the trial.

Fixed and adaptive arms: All lead clinicians from the 11 practices in the adaptive sub-group were interviewed at 3 months due to lower than predicted software use. No specific problems in using the software were identified. All lead clinicians felt low use reflected low patient demand. There was no difference in software use between fixed and adaptive practices at 12 months: fixed arm, mean 7.8 (s.d., 4.7) uses per practice per year per 10 000 registered patients; adaptive arm, mean 8.8 (s.d., 4.1); mean difference 0.9; 95% CI −2.8–4.8; *P*=0.60). We therefore combined data from the fixed and adaptive sub-groups of the intervention for all subsequent analyses.

**Figure 2:**
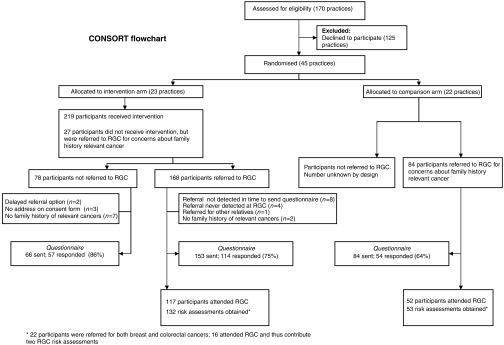
CONSORT Flowchart.

Practitioner confidence and attitudes: Lead clinicians' confidence in managing people with a family history of cancer increased significantly after training and this increase was maintained at 12 months (Figure 2). Their attitudes towards the software were generally positive, such that it was felt to be simple, easy, beneficial and cost-effective and these positive attitudes remained at 12 months (Figure 2). However, there was some reduction over time, in agreement with the statement that the software enhanced consultations (mean score 2.1 (s.d., 0.8) post-training; 3.0 (s.d., 1.7) at 12 months; mean change 0.8 95% CI 0.1–1.6; *P*=0.04; *n*=26) and persistent agreement that it would prolong consultations (mean score 2.5 (s.d., 1.2) post training; mean score 2.3 (s.d., 1.2) at 12 months). All but one lead clinician intended to continue using the software if it remained available. Median consultation time with the lead clinician was 28 min.

#### Patient level

A total of 219 patients received the GRAIDS intervention, of whom, 141 were referred to the Regional Genetics Clinic. A further 27 patients were referred to the Regional Genetics Clinic from GRAIDS practices without a GRAIDS consultation. Of the 168 referrals, 162 were identified at the Regional Genetics Clinic and were for relevant cancers. The 78/219 patients not referred were identified by the consent procedures. Risk perception was significantly lower in patients not referred to the Regional Genetics Clinic than in those referred.

## DISCUSSION

Practice team access to a family history assessment strategy using the GRAIDS software resulted in increased referral rates from primary care to a regional genetics clinic for familial breast and colorectal cancer, compared with current best practice. Referrals from GRAIDS practices were more appropriate than from comparison practices, when judged by their consistency with referral guidelines, the most relevant measure of general practitioners' clinical behaviour. Cancer worries were lower among patients referred from GRAIDS practices than from comparison practices. For the GRAIDS arm, patients who were not referred had lower cancer risk perception than those who were. Clinicians were generally positive about the software and intended to continue to use it if available.

### Limitations of design

Cluster randomisation at practice level to intervention and comparison strategies is the design of choice when the intervention is applied at that level (Donner, 2000). It avoids contamination between arms, which can reduce differences observed, but can pose problems in equivalence of recruitment and consent across arms. Thus we recruited patients in the GRAIDS arm as they consulted their GP. However, we wished in the comparison arm to mimic as closely as possible routine best practice (Watson *et al*, 2002). We therefore did not recruit patients who expressed concerns about their family history of cancer in this arm, to avoid an intervention effect by, for example, raising patient expectation and increasing clinician referrals independent of usual best practice. Consent to data collection in the comparison arm was seen only among those referred to the regional clinic. This meant that we were unable to collect data on patients in comparison practices, who presented but were not referred to the Regional Genetics Clinic.

The design precluded direct comparison of practice-based recruitment rates in the two arms; we therefore analysed referrals standardised by practice-registered population, where appropriate. Differences in principal referral outcomes between arms are thus an unbiased estimate of differences between the two overall service models, except for the timing of consent, the effect of which we believe would be small and not create any systematic bias.

### Key findings

This is the first report of a clinical trial demonstrating the value of family history assessment software designed for general practice. The trial design accounted for many of the recognised flaws in past trials of computer decision support systems (Mitchell and Sullivan, 2001). A previous study of mailing a CD Rom containing electronic guidelines for familial breast cancer to general practitioners in Scotland found minimal uptake of the software (Wilson *et al*, 2005). Our intervention differed in several critical ways: the software had greater utility by supporting collection of family history information and creating a pedigree; it made patient-specific recommendations about management at the point of decision; clinicians received interactive training in its use and the service model of training a single clinician in a practice led to more frequent use than if all practitioners had been trained (Kawamoto *et al*, 2005). The trial was testing the service model of providing a risk assessment service in the practice by supporting a single clinician in the practice regardless of background knowledge or interest. In some practices the lead clinician may have had a specific interest in familial disease or computer support, although to our knowledge this was only true in one practice. Over a quarter of practice teams approached were able to participate, a recruitment rate consistent with similar primary care trials (Montgomery *et al*, 2000). Practices recruited into the trial are likely to reflect the teams of the future who will be leading the incorporation of genetic medicine into practice (Rogers, 1995).

The GRAIDS intervention resulted in significantly more referrals that were consistent with referral guidelines, for breast cancer alone and combined with colorectal cancer. This difference was not apparent when appropriateness of referral was judged on the final Regional Genetics Clinic risk assessment; indeed the reverse was seen for colorectal cancer. This may reflect differences in the validity of self-reported family history of cancer across disease sites, which is usually confirmed by the genetics clinic (Ziogas and Anton-Culver, 2003). More importantly it reflects differences in the complexity and accuracy of the guidelines. The familial breast cancer guidelines are more complex than for colorectal cancer with several more criteria that define moderate and high-risk groups. Consequently they are more specific, but more difficult to implement in general practice. Computer implementation of familial breast cancer guidelines is more likely therefore to have a positive effect. The familial colorectal cancer guideline attempted to account for additional cancers associated with hereditary non-polyposis colon cancer (HNPCC), while still capturing individuals at moderate risk, and were therefore less specific. As a result, when applied rigidly by the software, practitioners made more referrals that were subsequently assessed as at population risk by the Regional Genetics Clinic, even though the referral would be considered ‘appropriate’, as it met the category for increased risk in the familial colorectal cancer guideline. This highlights the need for accurate guidelines for familial colorectal cancer to underpin decision support in primary care, similar to those published for breast cancer (McIntosh *et al*, 2004). Current criteria to identify individuals with potential HNPCC, such as the modified Bethesda criteria, aim to identify only high-risk individuals, and would fail to identify those at moderately increased risk, who may also benefit from referral (Umar *et al*, 2004).

Accurately identifying patients who may be at increased risk of breast and colorectal cancer, and reassuring the majority who are unlikely to benefit from referral is an important outcome of this trial. Although it resulted in increased referrals, which in the United Kingdom might threaten overstretched regional genetics services, it is the role of primary care to identify patients who might benefit most from specific referral, in this case for genetic counseling (Hayflick *et al*, 1998). There is growing evidence of benefit for a range of surveillance and prophylactic measures for individuals at increased risk of breast and colorectal cancer (Cuzick *et al*, 2003; Dove-Edwin *et al*, 2005; Leach *et al*, 2005). Access to predictive genetic testing can inform patients' decisions about prophylactic surgery and other preventive strategies (Meijers-Heijboer *et al*, 2000).

Patients referred from intervention practices reported significantly lower cancer worry than those from control practices. This was not mediated by risk perception or the knowledge items we measured. It is possible that the longer and more detailed assessment in primary care better prepared patients for referral with consequent reduction in anxiety. Alternatively, the GRAIDS intervention may have resulted in a different population referred to the Regional Genetics Clinic, who would not usually have been referred. Participants who were not referred from intervention practices showed lower cancer worry and mean risk perception than those who were referred. While acknowledging the absence of baseline data, this suggests that the intervention helps clinicians to reassure patients at population risk of familial cancer and manage them in primary care.

The intervention increased GPs' confidence in managing familial cancer, an area of medicine that is relatively new and complex from a primary care perspective (Emery *et al*, 2001). Clinicians were generally positive about the software in terms of simplicity and utility. However, concerns were evident about the time taken to conduct a consultation using the software. At the time of the trial, the majority of practices had limited bandwidth available via their NHSnet connection, making the software relatively slow to run. Recent expansion of broadband connections to practices in the UK would reduce the consultation time. However, this type of consultation cannot be conducted in a ‘standard 10 min’, as is true for a growing number of complex conditions managed in primary care (Freeman *et al*, 2002). What may be required is a brief, sensitive triage tool that identifies people with a family history of cancer and other common familial conditions for a more detailed assessment using GRAIDS-based software.

There is growing interest in the broader application of the family history of common disease in preventive health (Yoon *et al*, 2003). The Center for Disease Control has recently developed a similar electronic family history tool for use in primary care that is undergoing evaluation (Yoon and Scheuner, 2003). This trial demonstrates the potential of the GRAIDS software to improve the management of familial cancer in primary care, assuming the accuracy of current risk assessment guidelines. The software is now being developed to implement validated risk-assessment guidelines for other common familial conditions, to support the broader management of the family history of common disease in primary care.

## Figures and Tables

**Table 1:** Eastern Region Familial Breast/Ovarian and Colorectal Cancer Guidelines

*Breast/ovarian cancer*	
*High risk criteria:*	
1.	Two relatives who are FDR of each other with breast cancer where average age of diagnosis is under 40 years.
2.	Three or more relatives who are FDR of each other with ovarian or breast cancer, where average age of diagnosis is under 60 years
3.	Four or more relatives who are FDR of each other with breast or ovarian cancer at any age
4.	One individual in family with breast and ovarian cancer
	
*Moderate risk:*	
1.	One female FDR with breast cancer <40 years
2.	One paternal female SDR with breast cancer <40 years
3.	One female FDR with bilateral breast cancer <60 years
4.	Two FDR/SDR with breast cancer <60 years or ovarian cancer any age
5.	Three FDR/SDR with breast or ovarian cancer any age
6.	One male FDR with breast cancer any age
	
*Colorectal cancer*	
1.	One affected FDR <45 years
2.	One affected FDR and 1 affected SDR on same side of family
3.	Two FDR (inc both parents)
4.	Three affected relatives any age
	
*‘Affected’ means diagnosed with either of the following:*	
a.	CRC, colorectal cancer: 3⩾ adenomatous polyps, one adenomatous polyp <60 years.
b.	HRC, HNPCC-related cancer: endometrium, ovary, gastric, small bowel, ureter, renal pelvis
There should be at least one CRC in the family.	

Abbreviations: CRC, colorectal cancer; FDR, first-degree relative; HRC, HNPCC-related cancer; SDR=second-degree relative.

**Table 2:** Characteristics of practices and participants in trial arms

	**Intervention practices**	**Comparison practices**
*Practice factors at baseline*		
Number	23	22
Mean list size (s.d.)	8787 (3840)	8718 (4614)
Mean number of patients aged 20–50 years (s.d.)	3881 (1747)	3843 (2136)
		
*Participant factors at baseline*		
No. in trial not referred to RGC	78	Unknown by design
No. relevant referrals detected at RGC	162	84
		
*No. (%) referred to RGC for family history of*		
Breast/ovarian cancer	86 (53)	60 (71)
Colorectal cancer	55 (35)	23 (27)
Both	21 (13)	1 (1)

**Table 3:** Proportion of referrals meeting guidelines and number of referrals for increased risk, as determined by RGC, by randomised group

	**Intervention**	**Control**	**Odds ratio (95% CI)**
*Proportions meeting referral guidelines*			
Breast	93% (99/107)	73% (44/60)	4.5 (1.6–13.1)
Bowel	99% (75/76)	92% (23/25)	6.5 (0.5–83.7)
Combined	95% (174/183)	79% (67/85)	5.2 (1.7–15.8)
			*P*=0.006
*Proportions with increased RGC risk level*			
Breast	77% (60/78)	70% (23/33)	1.4 (0.6–3.5)
Bowel	56% (30/54)	85% (17/20)	0.2 (0.1–0.8)
Combined	68% (90/132)	75% (40/53)	0.7 (0.3–1.5)
			*P*=0.35

Abbreviations: CI, confidence interval.

Odds ratios and 95% confidence intervals shown for intervention *vs* comparison, allowing for the cluster randomised design.

NB: 21 participants were referred for a family history of breast and colorectal cancer, of whom 15 attended the RGC; these participants contribute two risk comparisons in the analyses.

**Table 4:** Patient knowledge, cancer worry and risk perception mean scores (standard deviations), and mean differences allowing for cluster randomised design

	**Intervention arm**				
	**Not referred**	**Referred**	**Mean difference**	**Comparison arm**	**Mean difference between referred populations (95% CI)**
Knowledge breast cancer	NA	5.77 (2.90)	NA	5.66 (2.78)	0.11 (−1.05–1.27)
		*n*=65		*n*=38	
Knowledge colorectal cancer	NA	5.50 (2.46)	NA	4.86 (3.30)	0.64 (−1.01–2.29)
		*n*=44		*n*=14	
Cancer worry	4.95 (2.99)	5.74 (3.04)	0.79 (−0.19–1.76)	7.18 (3.43)	−1.44 (−2.64 to −0.23)^*^
	*n*=57	*n*=110		*n*=51	
Risk perception	4.25 (0.80)	4.99 (1.14)	0.74 (0.38–1.09)^**^	5.04 (0.88)	−0.09 (0.34 to −0.51)
	*n*=51	*n*=104		*n*=47	

Abbreviations: CI, confidence interval; NA, not analysed since no hypothesised difference. ^*^P=0.02; ^**^P<0.0001.

**Table 5:** Accuracy of patients' risk perception compared with Regional Genetics Clinic assessment

	**Under-estimator**	**Accurate assessor**	**Over-estimator**
Comparison	9 (23%)	22 (55%)	9 (23%)
Intervention	18 (21%)	59 (68%)	10 (11%)
