# Supplementary figures and images for: The GRAIDS Trial: a cluster randomised controlled trial of computer decision support for the management of familial cancer risk in primary care
Source: Br J Cancer. 2007 Aug 14;97(4):486–93. doi: 10.1038/sj.bjc.6603897 (PMC2360348; doi:10.1038/sj.bjc.6603897)

Supplementary Figure 1 for online publication only: Screenshots of GRAIDS software


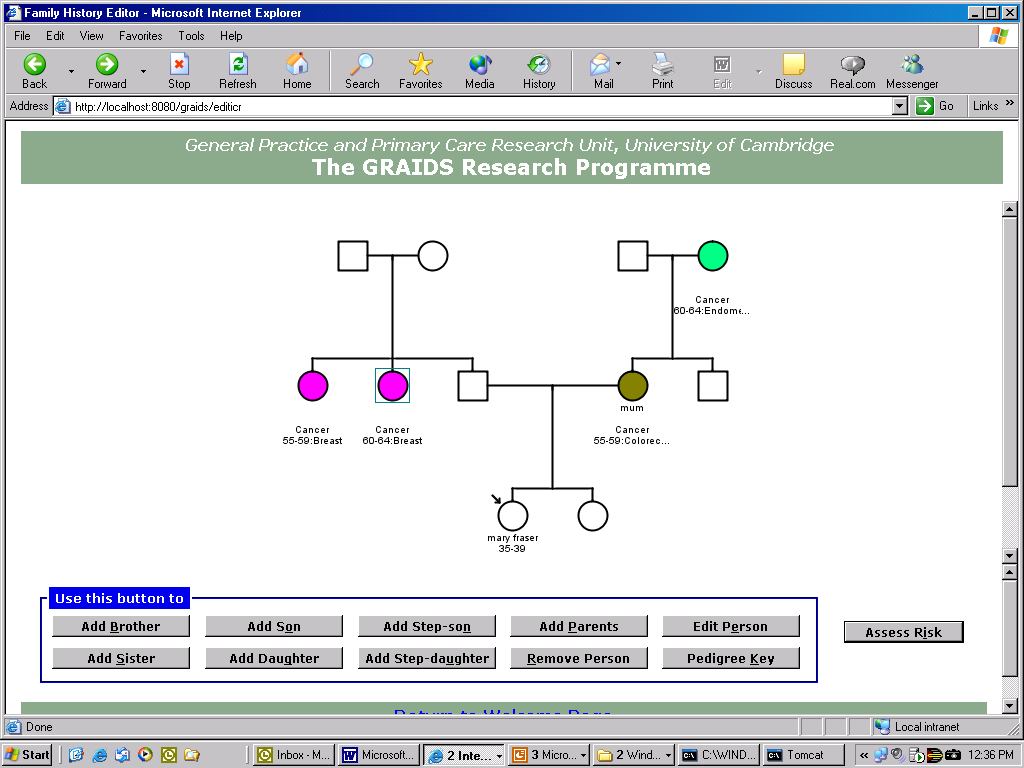


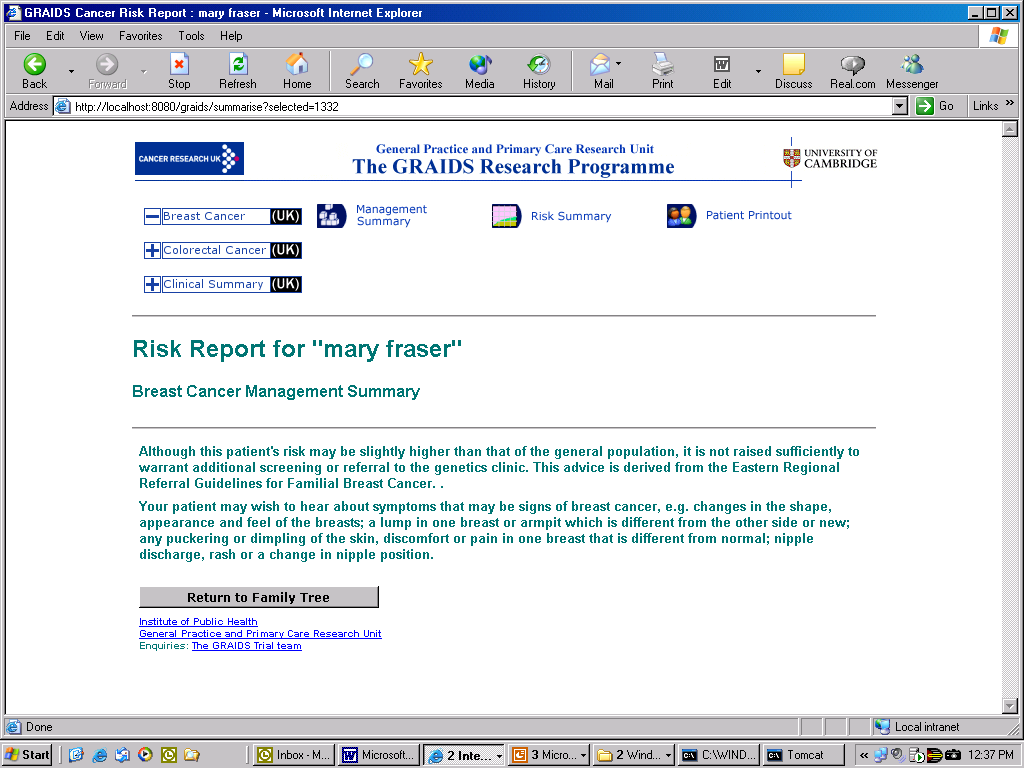


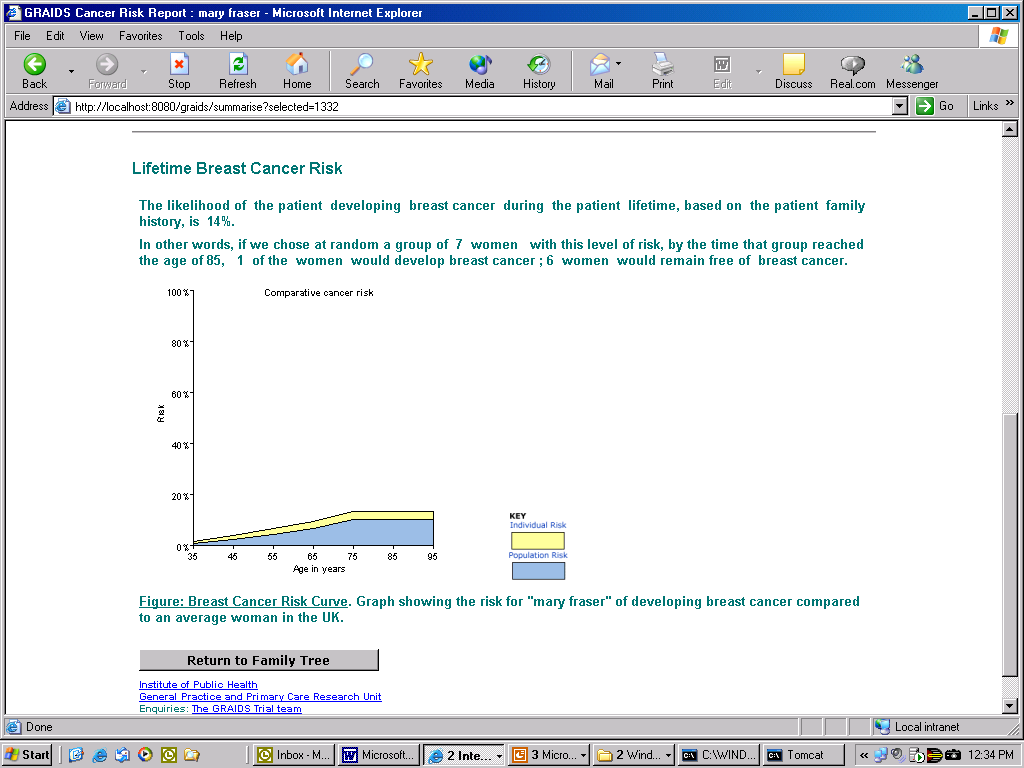

Supplement: Supplementary Figure 1 [file 6603897x1.doc]
